# Supplementary material for: Dynamic Distribution of Infectious Pancreatic Necrosis Virus (IPNV) Strains of Genogroups 1, 5, and 7 after Intraperitoneal Administration in Rainbow Trout (Oncorhynchus mykiss)
Source: Viruses. 2022 Nov 25;14(12):2634. doi: 10.3390/v14122634 (PMC9784894; doi:10.3390/v14122634)
Supplement: Supplementary file 1 [file viruses-14-02634-s001.zip › Table S1.pdf]

Table S1 The primers for amplifying IPNV- P202019 genome

Name	Primer sequence (5′→3′)		Product size (bp)
A1	Forward:	GGAAAGAGAGTTTCAACGTT	1598
	Reverse:	GTGTTAGGTAGCGGCCTCCG	
A2	Forward:	GCACCACTCATCGGAGCCGC	1568
	Reverse:	TGGGGGGCCGGGGGTTGAGG	
A3	Forward:	GGAAAGAGAGTTTCAACGTT	303
	Reverse:	TCCAGGAGCTCCAGGGAAGC	
A4	Forward:	GAGATCACAGACTTCTCAAG	297
	Reverse:	GTTGTTTGACTCCAGCCGTT	
A5	Forward:	AGAACCTGCACCAGAGGAAT	279
	Reverse:	TGGGGGGCCGGGGGTTGAGG	
B1	Forward:	TGGCACCCGACATACCACGA	1594
	Reverse:	CCATGAACTCTTTCGTCATG	
B2	Forward:	TCTCAACAACCACCTAATGT	1227
	Reverse:	TTATCTAGGGTCATGTTGGT	
B3	Forward:	TGGCACCCGACATACCACGA	303
	Reverse:	CATCTGTCTCTAGTTCGACC	
B4	Forward:	GACCTTCGCCATGAACGTCG	302
	Reverse:	CAGGTAGCCGTCCTGGGGCG	
B5	Forward:	GAAAAGAATCCAGTCACCGC	247
	Reverse:	TTATCTAGGGTCATGTTGGT	
