# Supplementary figures and images for: Dynamic Distribution of Infectious Pancreatic Necrosis Virus (IPNV) Strains of Genogroups 1, 5, and 7 after Intraperitoneal Administration in Rainbow Trout (Oncorhynchus mykiss)
Source: Viruses. 2022 Nov 25;14(12):2634. doi: 10.3390/v14122634 (PMC9784894; doi:10.3390/v14122634)

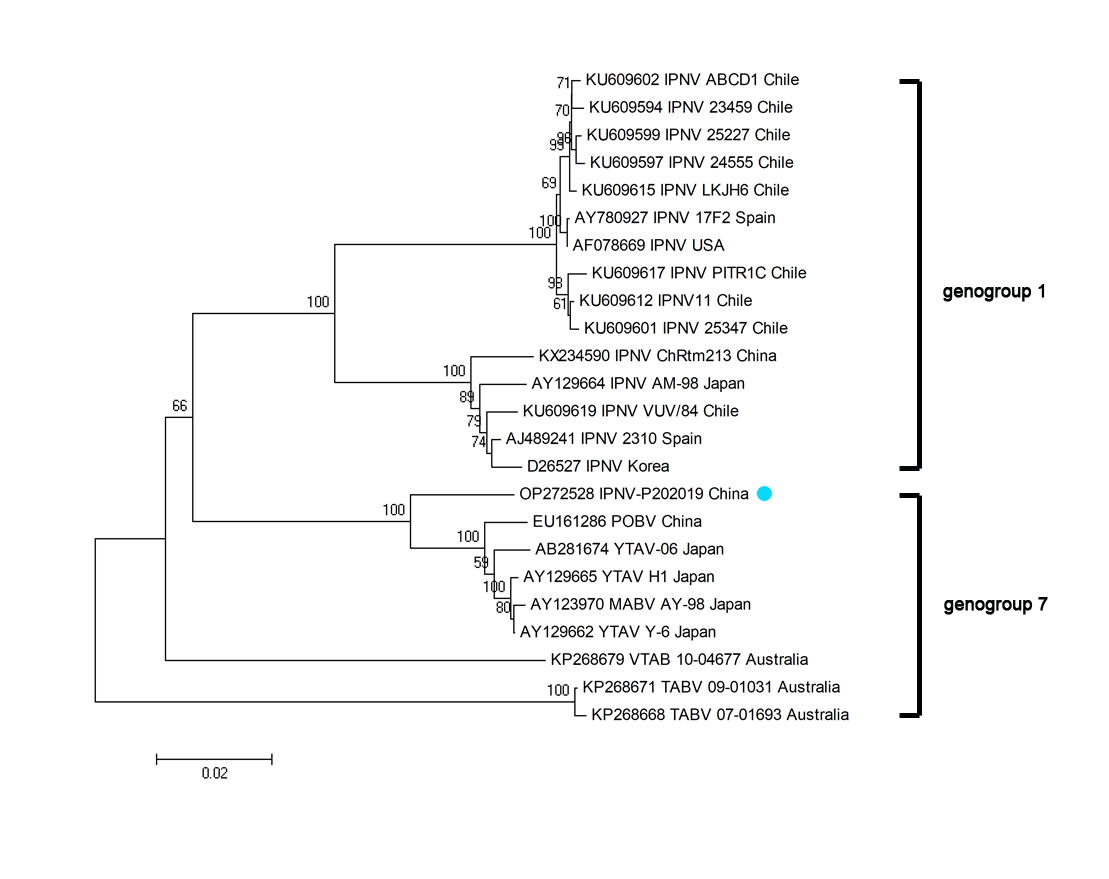

Supplement: Supplementary file 1 [file viruses-14-02634-s001.zip › Figure S1.tif]
